# Multidimensional Ln-Aminophthalate Photoluminescent Coordination Polymers

**DOI:** 10.3390/ma14071786

**Published:** 2021-04-04

**Authors:** Carla Queirós, Chen Sun, Ana M. G. Silva, Baltazar de Castro, Juan Cabanillas-Gonzalez, Luís Cunha-Silva

**Affiliations:** 1 LAQV/REQUIMTE & Department of Chemistry and Biochemistry, Faculty of Sciences, University of Porto, 4169-007 Porto, Portugal; carla.queiros@fc.up.pt (C.Q.); ana.silva@fc.up.pt (A.M.G.S.); bcastro@fc.up.pt (B.d.C.); 2 Madrid Institute for Advanced Studies, IMDEA Nanociencia, Calle Faraday 9, Ciudad Universitaria de Cantoblanco, 28049 Madrid, Spain; chen.sun@imdea.org

**Keywords:** photoluminescence, isophthalate, lanthanides, coordination polymers

## Abstract

The development of straightforward reproducible methods for the preparation of new photoluminescent coordination polymers (CPs) is an important goal in luminescence and chemical sensing fields. Isophthalic acid derivatives have been reported for a wide range of applications, and in addition to their relatively low cost, have encouraged its use in the preparation of novel lanthanide-based coordination polymers (LnCPs). Considering that the photoluminescent properties of these CPs are highly dependent on the existence of water molecules in the crystal structure, our research efforts are now focused on the preparation of CP with the lowest water content possible, while considering a green chemistry approach. One- and two-dimensional (1D and 2D) LnCPs were prepared from 5-aminoisophthalic acid and Sm^3+^/Tb^3+^ using hydrothermal and/or microwave-assisted synthesis. The unprecedented LnCPs were characterized by single-crystal X-ray diffraction (SCRXD), powder X-ray diffraction (PXRD), Fourier transform infrared (FT-IR) spectroscopy and scanning electron microscopy (SEM), and their photoluminescence (PL) properties were studied in the solid state, at room temperature, using the CPs as powders and encapsulated in poly(methyl methacrylate (PMMA) films, envisaging the potential preparation of devices for sensing. The materials revealed interesting PL properties that depend on the dimensionality, metal ion, co-ligand used and water content.

## 1. Introduction

Luminescence phenomena include photoluminescence (PL) and chemiluminescence, with fluorescence and phosphorescence being particular examples of PL. The photoluminescent properties of the materials essentially depend on: (i) fluorescence/phosphorescence intensity, (ii) energy absorption, (iii) photon absorption associated to electromagnetic radiation and (iv) nonradiative relaxation processes [1]. In the case of coordination polymers (CP), PL is also dependent on the ligand selected for the preparation of the materials. Considering these facts, a rational selection of the ligand can promote an efficient absorption and transfer of energy to an excited level of the Ln metal centers, leading to a sensitization of the Ln ions-antenna effect—corresponding to an increase in luminescence efficiency [2,3,4,5]. This occurrence allows the increase of the luminescence intensity of inorganic salts, generally limited by the low absorbance associated with forbidden *f*–*f* transitions.

Lanthanide-based coordination polymer (LnCP) luminescence, among other properties, can be improved or changed: using selected organic ligands, such as aromatic carboxylates [6,7,8,9,10,11], or introducing co-ligands, such as phenanthroline, bipyridine, 1,2-bis(4-pyridyl)ethylene and others [12,13,14,15,16,17,18]. These strategies are extremely important when the LnCP is prepared using aqueous solutions, since they tend to promote the replacement of water molecules present in the CP structure by the ligands [19,20], decreasing the effect of luminescence quenching associated to the presence of such molecules [3,21,22]. Several CPs based on isophthalic derivatives and Ln ions have been reported recently with different applications, namely detection of Fe^3+^ [15,23], Cu^2+^, CrO_7_^2−^, nitrobenzene [24], 2,4-dinitrophenol [23], antibiotics [25], alkylamines [26], as well as adsorption and removal of F^−^ from water [27]. Some of those studies focus on strategies to improve the PL properties. The work reported by Yan et al. revealed that an isophthalic acid-based metal-organic framework (MOF) was able to sensitize the luminescence of Sm^3+^ and Dy^3+^ materials despite the presence of several water molecules in the structure [28]. Another recurrent strategy is the use of doping agents, such as Eu^3+^ or Tb^3+^, to increase the emission intensity related to metal-centered emissions [29,30,31,32]. Kyprianidou et al. [33] describe that the replacement of solvent molecules by terminally coordinating organic molecules with multiple functional groups, including –OH, –SH, –NH– and –NH_2_, can increase up to 16-times the value of emission quantum yield. The antenna effect was observed in a Tb-based CP with 5-(1H-pyrazol-3-yl)isophthalic acid as a ligand, and this CP revealed high selectivity and sensitivity in the detection of PO_4_^3−^ in aqueous solution [34].

**Scheme 1:**
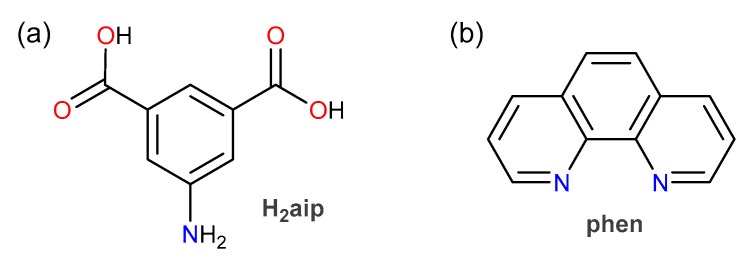
Chemical structure of the ligands used in the preparation of the CPs: (**a**) 5-aminoisophthalic acid (H_2_aip) and (**b**) phenathroline (phen).

The multiple examples of CPs prepared using isophthalic acid derivatives and their versatile application, combined with our recent research interests in the design and development of multifunctional crystalline materials [35,36,37,38,39,40,41,42,43], justify our motivation in the preparation of a series of LnCPs. Herein, four LnCPs, based on 5-aminoisophthalic acid (H_2_aip^−^, Scheme 1), prepared by hydrothermal (HT) or microwave-assisted synthesis (MWAS) methods, are reported. Considering that the PL properties of these materials are highly dependent on several factors, including the existence of water molecules in the crystal structure, our main objectives are the preparation of CP using a green chemistry approach, as well as the evaluation the influence of water content and structural differences in the PL properties of these materials. The PL properties of the LnCPs were studied in the solid state, at room temperature, including the determination of fluorescence intensity, PL excitation and lifetime, using the powders and a PMMA—poly(methyl methacrylate—encapsulation method.

## 2. Materials and Methods

### 2.1. Materials

Reagents and solvents used in the preparation of CP were purchased as reagent-grade and used without further purification, unless otherwise stated. MWAS was carried out in a circular single-mode cavity instrument (300 W max magnetron power output) from CEM (CEM Microwave Technology Ltd., Buckingham, UK).

For the PL characterization, spectrochemical grade solvents were used for optical measurements. Poly(methyl methacrylate) (PMMA) was obtained from Sigma-Aldrich (Darmstadt, Germany) and toluene was purchased from Merck Millipore (Darmstadt, Germany) without further purification.

### 2.2. Coordination Polymers’ Preparation

The CPs **SmCP1**, {[Sm(Haip)(aip)(H_2_O)_5_]⋅4H_2_O}*_n_*, and **TbCP1,** {[Tb(Haip)(aip)(H_2_O)_5_]⋅4H_2_O}*_n_,* were prepared by two distinct synthetic procedures, namely hydrothermal (HT) synthesis and microwave-assisted synthesis (MWAS), while the CPs **TbCP2**, {[Tb(Haip)(aip)(H_2_O)_2_]⋅H_2_O}*_n_*, and **TbCP3**, {[Tb(Haip)(aip)(phen)]⋅H_2_O}*_n_*, were only prepared by HT synthesis.

In general, the HT synthesis to obtain **SmCP1**, **TbCP1**, **TbCP2** and **TbCP3** was performed by the following procedure (the detailed description is presented in the Supplementary Material: Section 1, coordination polymers preparation): a mixture of 5-aminoisophthalic acid (H_2_aip, 1.0 equivalent), SmCl_3_⋅6H_2_O or TbCl_3_⋅6H_2_O (ca. 0.65 equiv), NaOH (1.0–1.2 equiv) and water (5 mL) was prepared in a 23 mL reaction vessel. For **TbCP1**, NaOH (1.3 equiv) was added and the pH was adjusted until 3 < pH < 4, while for **TbCP2**, NaOH (1.0 equiv) was added without adjustment of pH. In the case of **TbCP3**, the co-ligand 1,10-phenanthroline (phen, 0.59 equiv) was added to the mixture. For all the materials, the resulting mixture was stirred at room temperature for 30 min and then the reaction vessel was sealed and heated to 110 °C for 72 h. After cooling to room temperature, the resulting solid was filtered, washed with water and dried in air. Suitable crystals for single-crystal X-ray diffraction (SCXRD) were collected and analyzed (see more details in Supplementary Material, Figure S1).

The MWAS method to obtain **SmCP1** and **TbCP1** was performed according to the following: a mixture of H_2_aip (1.0 equiv), SmCl_3_⋅6H_2_O or TbCl_3_⋅6H_2_O (ca. 0.65 equiv), NaOH (1.1–1.2 equiv) and water (5.0 mL) was stirred at room temperature for 30 min in a closed 10 mL microwave vessel. The vessel was then placed in the cavity of the microwave reactor. The reaction mixture was irradiated at 110 °C (1 min ramp to 110 °C and 2 h at 110 °C, using 90 W maximum power, and the mixture was maintained with stirring). After cooling to room temperature, the resulting solid was filtered, and the filtrate was left standing at room temperature to obtain crystalline materials suitable for SCXRD analysis.

### 2.3. Single Crystal X-Ray Diffraction

Suitable single crystals of the material **SmCP1**, **TbCP1**, **TbCP2** and **TbCP3** were collected from the respective crystallization vial, immediately immersed in highly viscous oil and mounted on CryoLoops [44]. Diffraction data were collected on a Bruker X8 APEX II CCD area-detector diffractometer (Mo K_α_ graphite-monochromated radiation, λ = 0.71073 Å) with the acquisition controlled by the APEX2 software package [45]. The temperature of acquisition, 150(2) or 180(2) K, was set up with a cryosystem by the Oxford Cryosystems Series 700 monitored by the interface Cryopad [46]. Collected images were processed using the software package SAINT+ [47] and the absorption effects correction was carried out by the multi-scan semi-empirical method implemented in SADABS [48]. The structure was solved using the algorithms implemented in SHELXT-2014 [49,50] and the non-H-atoms were located from different Fourier maps calculated from successive full-matrix least squares refinement cycles on *F^2^* using SHELXL-v.2014 [49,51].

All the non-H-atoms were effectively refined by using anisotropic displacement parameters. H-atoms bound to carbon were placed at geometrical positions using suitable *HFIX* instructions in SHELXL and included in subsequent refinement cycles in riding-motion approximation with isotropic thermal displacements parameters (*U*_iso_) fixed at 1.2 × or 1.5 × *U_eq_* of the carbon atom to which they are attached. H-atoms of the numerous coordinated and uncoordinated water molecules were located from different Fourier maps and included in subsequent refinement stages, with the O···H and H···H distances restrained to assure an accurate geometry and using a riding-motion approximation with an isotropic thermal displacement parameter fixed at 1.5 × *U_eq_* of the respective O-atom.

Selected crystal and structure refinement data for the CP are summarized in Table 1.

### 2.4. Materials Characterization

Powder X-ray diffraction (PXRD) patterns were collected at room temperature using a Rigaku SmartLab diffractometer (Cu K*α*1,2 radiation, *λ*1 = 1.540593 Å and *λ*2 = 1.544414 Å) equipped with a D/teX Ultra 250 silicon strip detector and zero-background sample holder in a Bragg-Brentano para-focusing optics configuration (45 kV, 200 mA). Intensity data were obtained by the step counting method (step: 0.01°) in continuous mode in the approximate range 3.0° ≤ 2*θ* ≤ 50°.

Fourier transform infrared (FT-IR) spectra were obtained neatly with a FT-IR Perkin Elmer Spectrum BX with an attenuated total reflectance (ATR) accessory in the 400–4000 cm^−1^ range.

Scanning electron microscope (SEM) images and electron dispersive X-ray spectroscopy (EDX) spectra were acquired at CEMUP (Center of Materials from University of Porto) using a FEI Quanta 400FEG ESEM/EDAX Genesis X4M (15 keV).

### 2.5. Photoluminescence Characterization

The samples were prepared using two different methods, powder and PMMA encapsulation. In the powder method, crystalline material of the CPs was used to fill the aluminum sample holder with 2 mm diameter hole, and glass cover slides placed to cover the CP. The cover slides and aluminum sample holder were fixed with Teflon. In the PMMA encapsulation, 1–2 mg of the CP was placed on cleaned quartz substrate. One drop of 10 mg/mL PMMA in toluene was dropped on the CP powder to immobilize the powder to the substrate while acquiring the photoluminescence (PL) spectra. The prepared films of CP were then dried in air.

PL and PL excitation spectra were recorded at room temperature in a spectrofluorimeter FluoroMax-4 Horiba Jobin Yvon). PL was detected under 90° and long-pass filters were employed to minimize stray light caused by scattering of the excitation light by the powder. Time-resolved PL measurements were performed with a passive Q-switch Nd:YAG laser (TEEM Photonics) as an excitation source, delivering pulses at 355 nm of 300 ps duration at 75 Hz repetition rate. The PL was wavelength-dispersed and filtered by an Acton Research SP2500 spectrometer (f = 500 mm) and subsequently detected with a PicoQuant PMA 06 thermoelectrically cooled hybrid photomultiplier connected to a Picoquant TimeHarp 260 time-correlated single photon counting and multichannel scaling board electronics with 1 ns resolution. The decay time fitting analysis was carried out using Fluofit software (professional version) from PicoQuant.

## 3. Results and Discussion

### 3.1. Coordination Polymers Preparation

The CP preparation was conceptualized to use greener methodologies, including water as solvent, avoiding washing and crystallization processes with organic solvents, and applying MWAS to allow a faster and more environmentally safe strategy in comparison with the HT synthesis. Since a high content of water molecules in LnCP affects their PL properties, some variations in the synthetic methods, particularly the use of a co-ligand, were employed to achieve lower water content. Two isostructural CPs based in Sm^3+^ and Tb^3+^, designated **SmCP1** and **TbCP1**, were prepared following HT and MWAS procedures and two other unprecedent Tb^3+^ CPs were isolated by HT synthesis (**TbCP2** and **TbCP3**; experimental conditions are summarized in Table 2). The preparation of **TbCP3** involved the use of a co-ligand, the phenanthroline (phen).

As revealed in Table 2, a temperature of 110 °C was maintained for all HT and MWAS syntheses. Notably, by using the MWAS process, the **SmCP1** and **TbCP1** materials were obtained in 2 and 6 h respectively, in contrast to the 72 h required in the conventional HT process, meaning that the MWAS allows significant shortening of reaction time and consequently low energy consumption. However, it was also verified that under the tested MWAS reaction conditions, the products’ yields were slightly lower than that obtained with HT. Most probably, these lower yields are not only directly related to the MW irradiation source, but rather to the lower reaction time and rapid cooling that decrease the production yield of the materials, which may not be beneficial for these Ln materials.

In order to study the influence of the pH in the preparation of these materials a specific amount of base (NaOH) was added, for each synthesis. For a set of HT reactions, using Tb^3+^ as a metal center, three pH ranges (3–4, 7–8 and 10–11) were evaluated. SCXRD analysis revealed that crystalline materials different from the ligand were obtained only using pH = 3–4, indicating that the pH needs to be acidic for the LnCP preparation using H_2_aip as a ligand.

All the materials were characterized by SCXRD, PXRD, SEM/EDX and FT-IR.

### 3.2. Structures of the Coordination Polymers

Suitable crystals of **SmCP1** for SCXRD analysis were obtained and collected from the filtered solids (HT synthesis and MWAS), as well as from the filtrate of the MWAS, after 24 h. In the case of **TbCP1**, the crystals were isolated from the filtered solids in both synthetic methods. The SCXRD analysis revealed three one-dimensional (1D) CPs, **SmCP1**, **TbCP1**, **TbCP3**, and one two-dimensional (2D) CP, **TbCP2**.

**Figure 1:**
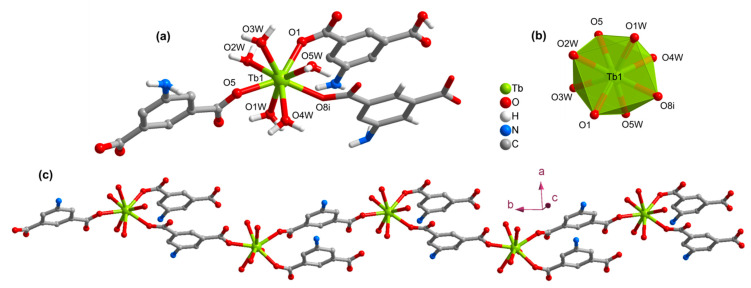
(**a**) Complete coordination environment for Tb1 center in the crystal structure {[Tb(Haip)(aip)(H_2_O)_5_]·4H_2_O}*_n_*, **TbCP1**, with the labelling scheme for all the atoms of the first coordination sphere (atoms of the asu are drawn in the ball-and-stick model and those generated by symmetry are shown in stick model). (**b**) Polyhedral representation of the Tb^3+^ coordination geometry center. (**c**) Neutral 1D CP observed in the crystal structure of **TbCP1**. Most of the H-atoms bonded were omitted for clarity. Symmetry transformation used to generate equivalent atom: (i) °*−x+3/2, °y−1/2, °−z+3/2*.

**SmCP1** and **TbCP1** crystal structures revealed the formula {[Ln(Haip)(aip)(H_2_O)_5_]⋅4H_2_O}*_n_*, with Ln = Sm^3+^ or Tb^3+^ for **SmCP1** or **TbCP1**, respectively. The two CPs are isostructural and crystallized in a monoclinic system (space group *P*2_1_/*n*) with the respective asymmetric unit (asu) containing a Ln^3+^ center, two coordinated organic ligands (one fully deprotonated, aip^2−^, and the other monoprotonated, Haip^−^), five coordinated and four uncoordinated water molecules (Figure 1a). The crystal structure description and discussion will be detailed and discussed only for the **TbCP1**, being similar and applicable for the Sm-based material (more information about the **SmCP1** crystal structure is shown in the Supplementary Material: Figure S2 and Table S1). The Tb^3+^ metal center is eight-coordinated, {TbO_8_}, bonding to three carboxylate O-atoms (O1, O5 and O8i) from three different ligands by unidentate coordination modes and five water molecules (O1W to O5W), in a distorted square antiprismatic geometry (Figure 1a,b). The Tb–O distances range from 2.338(4) to 2.426(4) Å, while the O–Tb–O angles show very large differences depending on the position of the O atoms in the polyhedral: minimum value of 70.77(17) for O5–Tb–O4W and maximum of 147.98(17) for O1–Tb–O4W (complete information about Tb–O distances and O–Tb–O angles is provided in the Supplementary Material, Table S2). The Haip^−^ ligand interconnects the two Tb^3+^ centers by unidentate bridging modes originating 1D CP (coordination chain) along the *b*-axis of the unit cell of **TbCP1** (Figure 1b and Figure S3 of the Supplementary Material). The additional connection of one aip^-^ ligand per Tb^3+^ center led to a neutral coordination chain, {[Tb(Haip)(aip)(H_2_O)_5_]}*_n_*, with Tb∙∙∙Tb distances of 11.741(1) Å.

**Figure 2:**
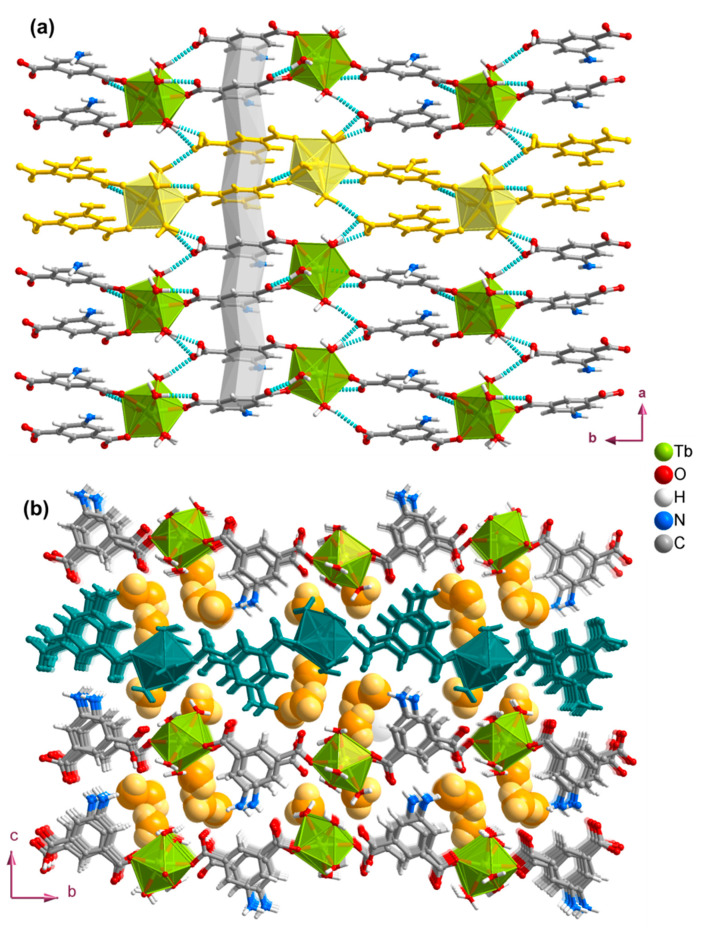
Structural features of the crystal structure of **TbCP1**: (**a**) two-dimensional (2D) supramolecular network extended in the [001] direction of the unit cell, with the O–H···O hydrogen bonds drawn as light-blue dashed lines and the *π-π* stacking interactions represented as a grey shadow (one of the coordination chains are shown in yellow color). (**b**) Extended packing arrangement viewed in the [100] direction of the unit cell with the crystallization water molecules occupying the cavities (these water molecules are drawn in a space fill model and orange/yellow color scheme).

The adjacent neutral coordination chains, {[Tb(Haip)(aip)(H_2_O)_5_]}*_n_*, in **TbCP1,** are engaged in an extensive network of strong O−H···O hydrogen bonds (light-blue dashed lines in Figure 2b; for geometric details of these intermolecular interactions see Table 3) originating a 2D supramolecular network (supramolecular layer) extended in the *ab* plan of the unit cell. The contiguous organic molecules within the layers, Haip^−^ and aip^2−^ ligands, are also involved in slight offset *π*−*π* stacking interactions, with the *Cg*···*Cg* distances of the phenyl rings of 3.425(4), 3.759(4), 3.469(4), 3.765(4) and 3.453(4) Å (grey shadow in Figure 2a; *Cg* represents the gravity center of the ring). Individual supramolecular layers closely pack along the [001] direction of the unit cell, creating some cavities filled by crystallization water molecules (Figure 2b). The structural cohesion of this crystalline packing arrangement is strengthened by an extensive network of O−H···O and N−H···O hydrogen bonds involving the adjacent layers and the crystallization water molecules, ultimately leading to a three-dimensional (3D) supramolecular framework (H-bonds not shown).

**Figure 3:**
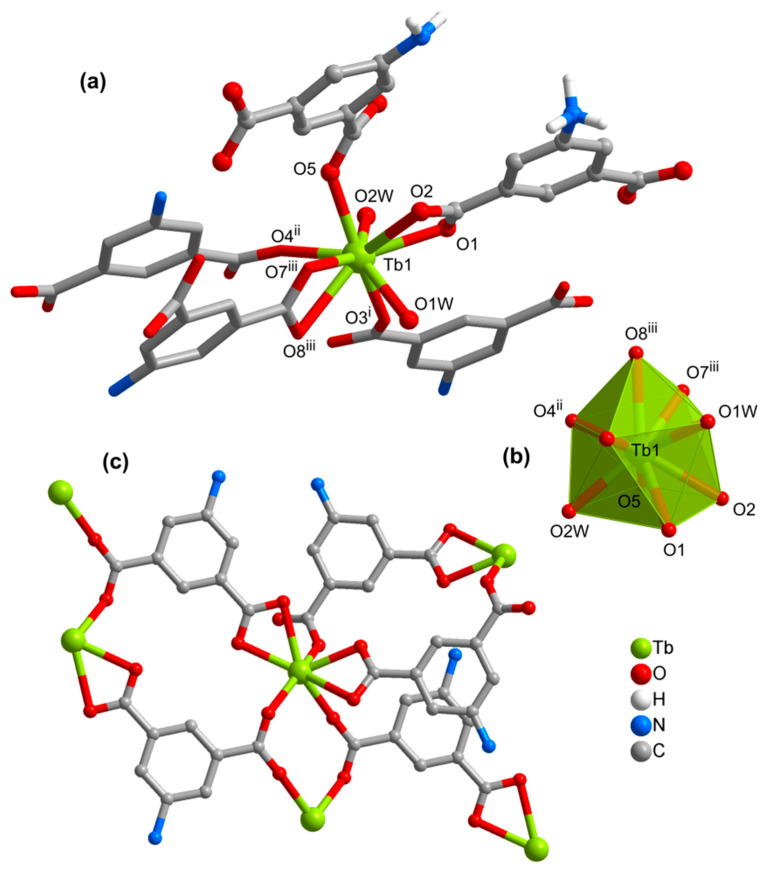
(**a**) Complete coordination environment for Tb1 center in the crystal structure of **TbCP2**, {[Tb(Haip)(aip)(H_2_O)_2_]·H_2_O}*_n_*, with the atoms of the asu drawn in the ball-and-stick model and those generated by symmetry shown in the stick model (for clarity, most of the H-atoms were omitted). (**b**) Polyhedral representation of the Tb^3+^ coordination geometry center. (**c**) Partial extended structure of the **TbCP2**. Symmetry transformations used to generate equivalent atoms: (i) *−x, −y+1, −z*; (ii) *x, y−1, z*; (iii) *−x+1, −y, −z*; (iv) *x, y+1, z*.

The crystal structure of **TbCP2** was determined in the monoclinic system, with space group *P*2_1_/*c*, and revealed a 2D CP (coordination layered material) with general formula {[Tb(Haip)(aip)(H_2_O)_2_]⋅H_2_O}*_n_*. Comparatively to the previous CPs, **SmCP1** and **TbCP1**, {[Tb(Haip)(aip)(H_2_O)_5_]⋅4H_2_O}*_n_*, this one revealed a significant decrease in the coordinated and uncoordinated water molecules: a total diminution of seven water molecules is verified. Furthermore, small variation in the reactant’s ratio led to a dimensionality increase: 1D for **TbCP1** to 2D for **TbCP2**. The complete coordination environment for Tb center in the crystal structures of **TbCP2** is shown in Figure 3a, being nine-coordinated {TbO_9_} and presents a geometry closer to a distorted mono-capped square antiprismatic (Figure 3b). Tb–O bond lengths were found between 2.290(3) Å and 2.661(3) Å, while the O–Tb–O angles ranged from 51.46(9)° to 155.01(10)° (more detailed information can be obtained in Table S3 of the Supplementary Material).

**Figure 4:**
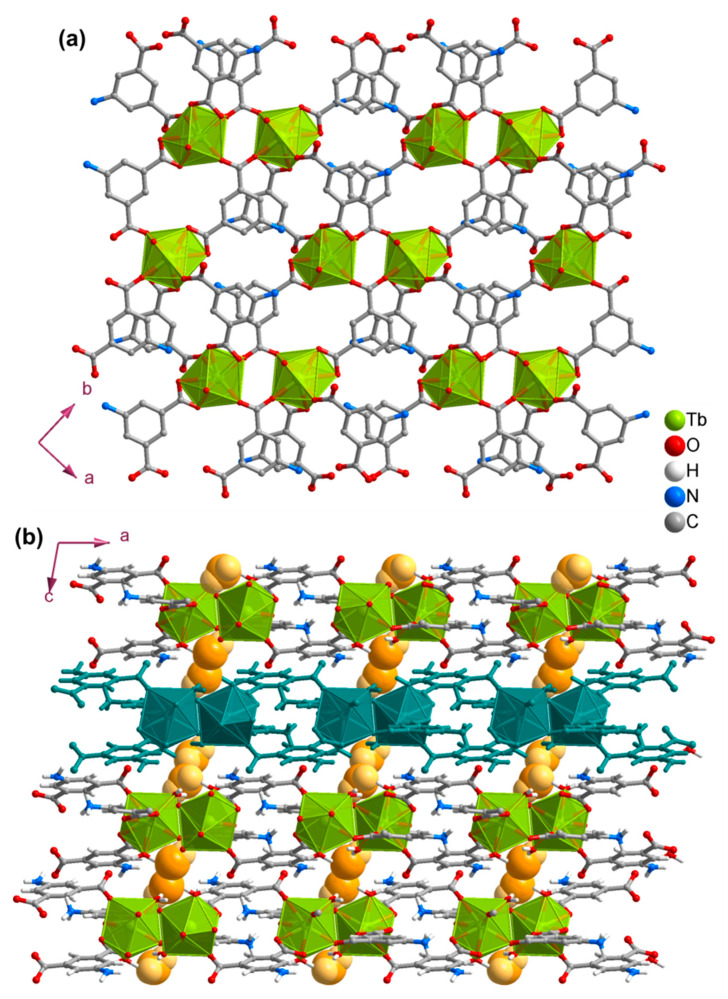
Structural features of the crystal structure of **TbCP2**, {[Tb(Haip)(aip)(H_2_O)_2_]·H_2_O}*_n_*: (**a**) neutral 2D CP (coordination layer), extended in the *ab* plan of the unit cell. (**b**) Close packing of individual coordination layers of along the [001] direction (one of the layers is shown in green color and the crystallization water molecules are drawn in a space fill model and orange/yellow color scheme).

The extended crystalline structure features reveal that the crystallographic independent aip^2−^ and Haip^−^ organic molecules behave as multidentate ligands due to the coordination with various Tb^3+^ centers (Figure 3c and Figure S4 at Supplementary Material). In fact, these ligands interconnect the Tb^3+^ centers by unidentate and bidentate bridging modes, leading to the formation of neutral 2D CPs (coordination layers): extended in the *ab* plane of the unit cell (Figure 4a). The layers pack along the [001] direction of the unit cell and small pores in the interlayer space, which is occupied with crystallization water molecules (Figure 4b). Furthermore, the close packing of individual layers is mediated by strong and highly directional interlayer O−H···O and N−H···O hydrogen bonds (not shown), ultimately creating 3D supramolecular structures (details about the H-bonds in Table 4).

**Figure 5:**
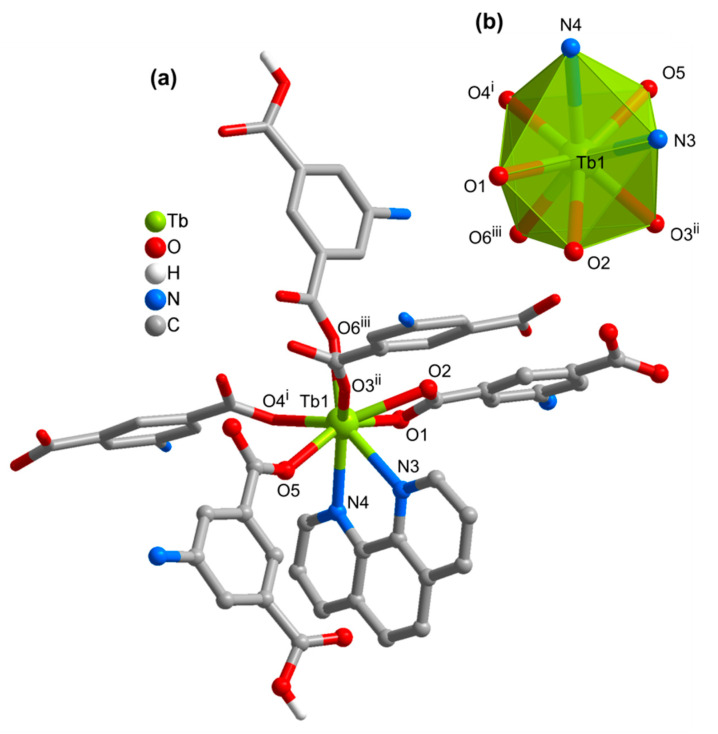
(**a**) Complete Tb1 coordination center in the crystal structure of **TbCP3**, {[Tb(Haip)(aip)(phen)]·H_2_O}*_n_*, showing the labelling scheme for all the atoms of the first coordination sphere (atoms of the asu are drawn in the ball-and-stick model and those generated by symmetry are shown in the stick model. H-atoms bonded to carbons were omitted for clarity). (**b**) Polyhedral representation of the Tb^3+^ coordination geometry center. Symmetry transformations used to generate equivalent atoms: (i) *−x,−y+1,−z+1*; (ii) *−x+1, −y+1 −z+1*; (iii) *x+1, y, z*.

The attempt to further decrease the CP water content by using the co-ligand phen resulted in a CP crystallized in a monoclinic system with the crystal structure solved in the space group *P*2_1_/*c*, **TbCP3**. The introduction of the phen in the crystalline structure also led to a decrease in the dimensionality of the CP, relatively to **TbCP2**, as previously reported in related CPs [12]. The asu of the crystal structure of **TbCP3** comprises a Tb^3+^ center, a fully deprotonated aip^2−^ ligand, a mono-protonated Haip^−^ ligand, a phen molecule and one water molecule (uncoordinated), unequivocally confirming the formula of the 1D coordination compound as {[Tb(Haip)(aip)(phen)]⋅H_2_O}*_n_* (Figure 5 and Figure S5 in the Supplementary Material). Looking at **TbCP2** and **TbCP3** formulas, it can be verified the exchange of the two coordinated H_2_O molecules in **TbCP2** for a phen molecule in **TbCP3**. Interestingly, despite the introduction of the phen ligand, the **TbCP3** revealed the identical coordination number of the Tb^3+^ center (eight) in **TbCP1**, and the same CP dimensionality (1D). Most probably, these similarities can be related with the identical synthesis conditions, despite the utilization of the additional co-ligand phen.

The Tb^3+^ center coordinates to six carboxylate *O*-atoms from three aip^2−^ ligands (two unidentate and one bidentate chelating coordination modes) and from two Haip^−^ ligands (two unidentate modes). The metal coordination sphere is completed by two *N*-atoms from the phen ligand, leading to an eight-coordinated center {TbO_6_N_2_} showing a distorted dodecahedral geometry (Figure 5b). The Tb–O bond lengths range from 2.277(4) to 2.453(4) Å, while Tb–N distances are considerably longer, 2.563(5) and 2.598(5) Å. The O(N)–Tb–O(N) angles are found between 63.51(16)° and 147.31(13)° (for additional details about these bond lengths and angles, please consult the Supplementary Material, Table S4).

**Figure 6:**
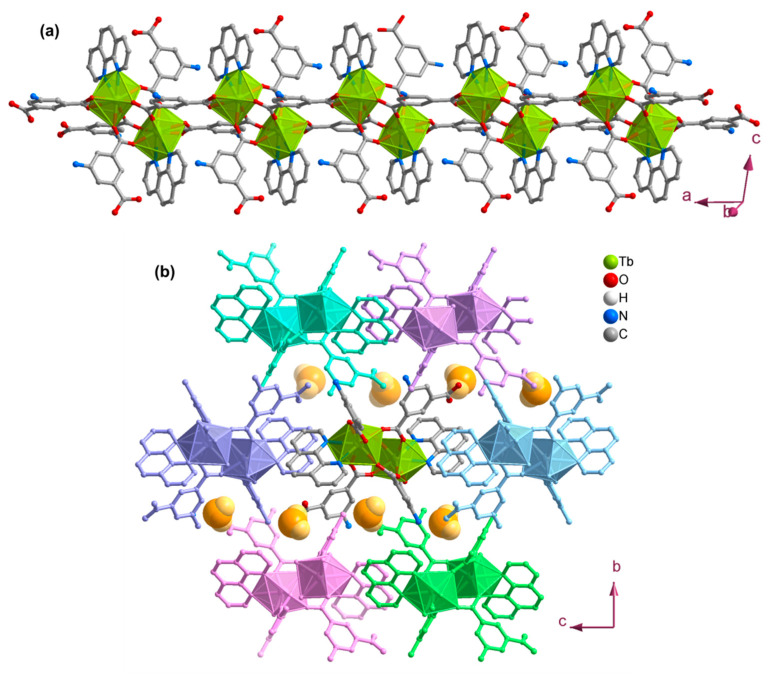
Structural features of the crystal structure of **TbCP3**, {[Tb(Haip)(aip)(phen)]·H_2_O}*_n_*: (**a**) 1D CP with ladder arrangement (coordination chain). (**b**) Extended packing viewed in the [100] direction of the unit cell, with the different coordination ladders drawn in distinct colors, and the crystallization water molecules depicted in the space fill model (orange/yellow color scheme). H-atoms, except for those belonging to water molecules, have been omitted for clarity.

In **TbCP3** crystal structure, the Haip^−^ and aip^2−^ ligands interconnect the Tb^3+^ centers by unidentate bridging and chelate modes (Supplementary Material: Figure S5b), promoting the formation of an interesting 1D CP (coordination chain) with a distorted ladder structure along the *a*-axis of the unit cell, and the phen ligands of adjacent metallic centers oriented in opposite directions (Figure 6a). Ligand arrangement imposes inter-ladder Tb···Tb distances of 4.270(1), 8.250(2), 10.350(2) Å and 13.514(3) Å. The neighboring 1D ladders are involved in an extensive network of strong N−H···O and O−H···O intermolecular interactions (not shown), leading to an overall crystalline packing arrangement with voids occupied by uncoordinated water molecules (Figure 6b; more details concerning the geometric information of the H-bonds can be consulted in Table 5).

### 3.3. Crystalline and Morphology Analysis

**Figure 7:**
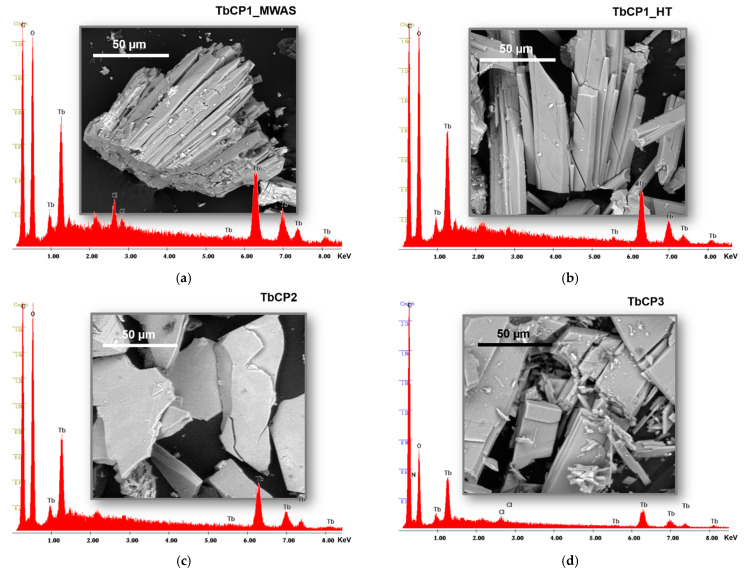
SEM images and respective EDX spectra for **TbCP1** prepared by MWAS (**a**) and HT (**b**), **TbCP2** (**c**) and **TbCP3** (**d**).

Scanning electron microscopy (SEM) characterization was performed for all Tb-based CPs, and the results are summarized in Figure 7, including the respective EDX spectra for each one of the materials. This analysis is important to evaluate the approximate size and morphology of the prepared CP materials and may also help in the assessment of the presence of organic/inorganic contaminants or other crystalline phases (EDX analysis).

**Figure 8:**
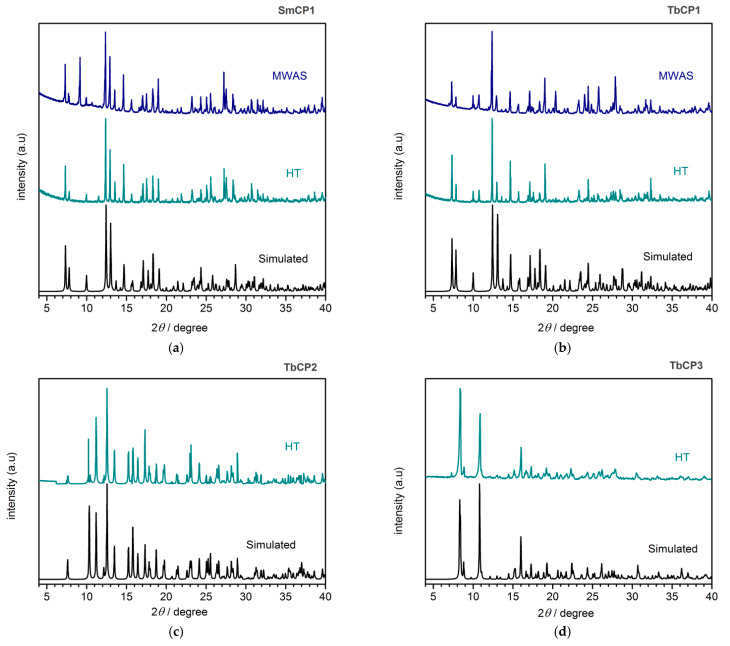
Powder X-ray diffraction (XRD) patterns the all the CP materials obtained experimentally and compared with the respective diffractogram obtained by simulation from the single-crystal XRD data: SmCP1 (**a**), TbCP1 (**b**), TbCP2 (**c**) and TbCP3 (**d**).

SEM analysis of **TbCP1** reveals that the material prepared by HT is more disperse and morphologically more homogeneous than the one prepared by MWAS, that shows agglomerates of long needles (Figure 7a,b). Most probably, these differences may be associated with a distinct crystallization process, faster in the case of MWAS considering that the cooling takes about 20 min instead of the several hours of the HT synthesis (promoted by the difference in the reaction vessels, glass for MWAS and Teflon/stainless reactor for HT). In general, a faster cooling promotes the preparation of materials of lower crystallinity and lower conversion into the desired CP. Despite this dissimilarity in the morphology of the **TbCP1** obtained by MWAS and HT synthesis, the powder XRD analysis confirmed that the crystalline materials isolated by the two methods have the same solid-state structure and it largely corresponds to that predicted from the single-crystal data simulation (Figure 8). However, the observation of a few additional peaks relatively to simulated, points to the residual presence of some additional crystalline phase or compound. Most probably, these additional peaks can correspond to some residual non-reacted TbCl_3_ salt, since the EDX spectra of all the CPs reveal the occurrence of Chloride.

As expected, the **TbCP2** and **TbCP3** reveal distinct crystalline morphology and particle size (Figure 7c,d, respectively). While the coordination layer material (2D CP **TbCP2**) shows large plate shape particles, the coordination chain material (1D CP **TbCP3**) comprises crystalline particles more elongated and with size variability. The powder XRD analysis of these CPs confirms that the bulk materials were isolated as a pure crystalline phase, since the experimental powder XRD patterns corresponds to the respective patterns simulated from the single-crystal data (Figure 8). All the EDX spectra unequivocally confirm incidence of the main elements of the CPs, namely Tb, C and O. In fact, the choice of the synthetic methods is an important factor for the control of size and morphology of the CP particles, which is also a fundamental issue regarding the potential applicability of the materials. Most of the previous discussion based in SEM/EDX and powder XRD is supported by the FT-IR spectra (Figure S6 in Supplementary Material).

### 3.4. Photoluminescence Studies

**Figure 9:**
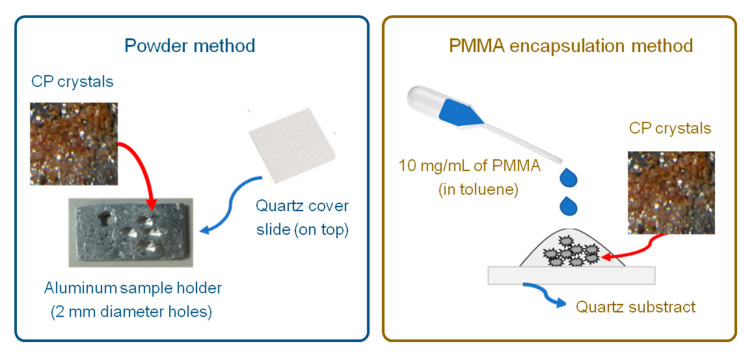
Schematic illustration of the powder (**left side**) and PMMA encapsulation (**right side**) methods used for the study of the PL properties.

The PL properties of Sm- and Tb-based CPs were investigated at room temperature in the solid state as powders or encapsulated in PMMA (Figure 9). In general, the results obtained by the two methods were similar, thus only the results obtained with the PMMA encapsulation method will be presented/discussed. The fluorescence intensity and PL lifetime parameters are summarized in Table 6, and the analysis of these results indicates that there are significant differences between all the CPs, demonstrating the effect of the crystalline structure features in the PL properties.

The PL properties of **SmCP1** and **TbCP1** prepared by HT and MWAS were similar between the two synthetic methodologies. Nevertheless, for **TbCP1** a decrease in the lifetime value of ≈20% is observed from HT to MWAS materials. The comparison of the lifetime values of **SmCP1** and **TbCP1** (Table 3)—4.43 μs vs. 185.57 μs (HT) and 4.39 μs vs. 148.15 μs (MWAS), respectively—indicates that the energy transfer from ligand, CP1, to Tb^3+^ metal center is much more efficient than to Sm^3+^ metal center. It is clear the importance of the selection of the metal center in the preparation of luminescent CP. Regarding **TbCP2** and **TbCP3**, only one set of data was collected since these materials were obtained using only HT synthesis. The “exchange” of the coordinated H_2_O molecules in **TbCP2** for a phen molecule in **TbCP3** led to a significant change in the PL properties, particularly in the lifetime value. The lifetime value of **TbCP3** is 6.8 times higher than that of **TbCP2**.

**Figure 10:**
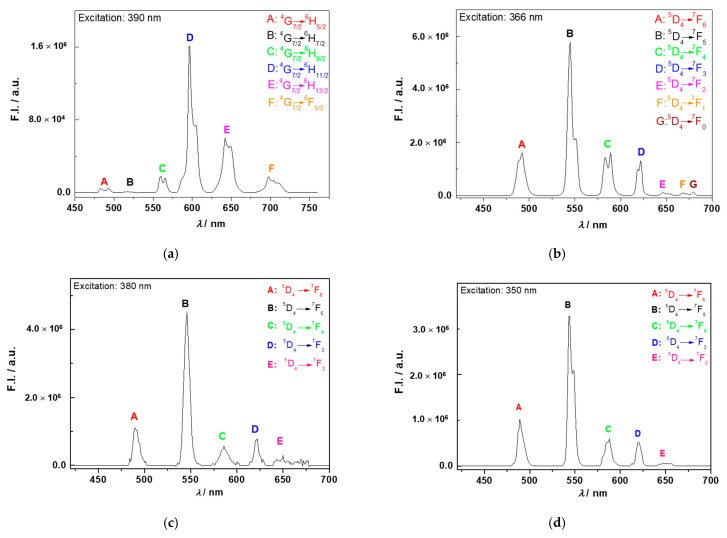
Graphical representation of the fluorescence intensity spectra of **SmCP1** (**a**), **TbCP1** (**b**), **TbCP2** (**c**) and **TbCP3** (**d**).

The Sm^3+^ CP showed relatively narrow emission peaks corresponding to the expected transitions associated with Sm^3+^ (Figure 10A). PL excitation spectra and the graphical representations of the PL intensity in function of lifetime are included in the Supplementary Material (Figure S7). All the Tb^3+^ CP showed identical narrow emission peaks (Figure 10B–D), with very small deviations in the emission wavelengths (Table 6), corresponding to the ^5^D_4_ →_7_F_J_ (J = 6–2) transitions of Tb^3+^ ion, with the maximum peaks at 545/543 and 546 nm respectively, which could be ascribed to the contribution from the ^5^D_4_ → ^7^F_5_ transition. The spectra and data presented are very similar to those reported by Sarma and co-workers [45], including those of lifetime values (approximately 100 μs) for the 1D CP. The more noteworthy difference between the CP luminescence spectra is the presence of two additional transitions for **TbCP1** relative to **TbCP3**, corresponding to the ^5^D_4_ →^7^F_J_ (J = 1–0) transitions of the Tb^3+^ ion; on the other hand, the resolution of the **TbCP2** spectrum does not allow for the observation of such transitions with certainty (the very noisy part of the spectrum is caused by the instrument of the exciting wavelength). The PL excitation spectra of **TbCP1** and **TbCP3** (Supplementary Material: Figure S8) revealed a broad band with a maximum at approximately 390 nm, which can be associated with the intra-ligand transitions of the H_2_aip ligand, as reported in related compounds [24,45]. The presence of the PL excitation band due to H_2_aip ligand can be proof for an efficient energy transfer (assuming that all the emissions at the wavelength of detection come from the Ln).

## 4. Concluding Remarks

A series of multidimensional LnCP (namely, **SmCP1**, **TbCP1**, **TbCP2** and **TbCP3**) was prepared using sustainable procedures, namely: (a) water was used as solvent, the use of organic solvents was avoided, and a fast crystallization process was achieved and (b) the MWAS process was found to be a suitable alternative for the preparation of **SmCP1** and **TbCP1** materials, by reducing the reaction time of 72 h to 2 or 6 h, respectively. A common feature of all the LnCP is the presence of several coordinated and uncoordinated water molecules, except for **TbCP3** that only contains one uncoordinated water molecule. In the case of **TbCP2** and **TbCP3**, the use of phen as a co-ligand led to the decrease in the CP dimensionality from 2D (**TbCP2**) to 1D (**TbCP3**), associated to eight- or nine-coordinated Tb^3+^ center, respectively.

The PL properties in the solid state were studied, employing two methods: powder and PMMA encapsulation, and the results were similar for both techniques. The PL properties study revealed the influence of the structural differences between the four LnCP. For all cases, the expectable transition bands, for each Ln, were observed. In some cases, like **SmCP1**, the PL excitation band associated to the H_2_aip ligand was present, possibly indicating a more efficient energy transfer from the ligand-to-Sm^3+^. Although, for **TbCP2**, the lower intensity bands and the presence of a small band at ≈325 nm could indicate that the ligand-to-Tb^3+^ de-activation occurs more efficiently in comparison to **TbCP1** and **TbCP3**. Also, the difference observed in the Tb^3+^ CP lifetimes may reflect a different environment surrounding the lanthanide ion, associated to the difference in the water content and presence of phen as a co-ligand. This first evaluation of the influence of synthetic methods in the PL properties of Ln-H_2_aip CP reveals a wide number of possibilities to evaluate and improve the PL properties of the materials. Interesting study approaches can include the modification of the reactions’ temperature and time, the removal by heat/vacuum of the H_2_O molecules of the CP frameworks (without destroying them) and post-functionalization with ligands containing different functional groups or co-ligands. The potential of these Ln-H_2_aip CPs can also be extended to other fields, such as heterogeneous catalysis or sensing capability towards solvent molecules, small molecules or metal ions.

## Figures and Tables

**Table 1:** Selected crystal and structure refinement data for the CPs: **SmCP1**, **TbCP1**, **TbCP2** and **TbCP3**.

	SmCP1	TbCP1	TbCP2	TbCP3
Formula	C_16_H_29_N_2_O_17_Sm	C_16_H_29_N_2_O_17_Tb	C_16_H_17_N_2_O_11_Tb	C_28_H_21_N_4_O_9_Tb
*Mr*	671.76	680.33	572.23	716.41
Crystal morphology	Colorless plates/prisms	Colorless needles	Pink prisms	Colorless plates
Crystal size/mm	0.18 × 0.07 × 0.05	0.19 × 0.05 × 0.04	0.33 × 0.10 × 0.08	0.19 × 0.06 × 0.04
Crystal system	Monoclinic	Monoclinic	Monoclinic	Monoclinic
Space group	*P*2_1_/*n*	*P*2_1_/*n*	*P*2_1_/*c*	*P*2_1_/*c*
*a*/Å	7.1362(2)	7.1046(8)	11.8098(8)	10.350(2)
*b*/Å	22.6821(6)	22.583(2)	10.7888(7)	21.289(5)
*c*/Å	14.2428(4)	14.2477(16)	14.3259(8)	12.490(3)
*β/*°	92.0350(10)	92.039(6)	100.253(2)	104.125(9)
Volume/Å^3^	2303.94(11)	2284.5(4)	1796.2(2)	2668.9(10)
*Z*	4	4	4	4
*ρ*_calculated_/gcm^-3^	1.937	1.978	2.116	2.714
Final R índices [I > 2*σ* (I)]	R_1_ = 0.0275 wR_2_ = 0.0568	R_1_ = 0.0494 wR_2_ = 0.0863	R_1_ = 0.0324 wR_2_ = 0.0611	R_1_ = 0.0476 wR_2_ = 0.0818
R índices (all data)	R_1_ = 0.0363 wR_2_ = 0.0611	R_1_ = 0.0958 wR_2_ = 0.1024	R_1_ = 0.0400 wR_2_ = 0.0634	R_1_ = 0.0906 wR_2_ = 0.0960

**Table 2:** Summary of the experimental parameters and conditions used in the synthesis of the four CPs.

CP	Equiv				Synthesis	T/°C	t/h	*η*/%
	H_2_aip	LnCl_3_.6H_2_O	NaOH	phen				
SmCP1	1.0	0.67	1.1	–	HT	110	72	28
		0.64	1.2		MWAS		2	21
TbCP1	1.0	0.64	1.2 *	–	HT	110	72	37
		0.67	1.1		MWAS		6	16
TbCP2	1.0	0.66	1.0	–	HT	110	72	27
TbCP3	1.0	0.66	1.2	0.59	HT	110	72	47

* Corresponding to the initial quantity weighted. The pH value of the reactional mixture was adjusted for 3–4 with addition of a 1 M NaOH aqueous solution.

**Table 3:** Geometric information (distances in Å and angles in degrees) for the D–H···O hydrogen bond interactions of **SmCP1**, {[Sm(Haip)(aip)(H_2_O)_5_]∙4(H_2_O)}*_n_*, and **TbCP1**, {[Tb(Haip)(aip)(H_2_O)_5_]∙4(H_2_O)}*_n_*, crystal structures.

SmCP1					TbCP1			
D–H···O	*d* (H···O)	*d* (D···O)	∠ (DHO)		D–H···O	*d* (H···O)	*d* (D···O)	∠ (DHO)
O1W–H1W···O5W^iii^	1.927(2)	2.801(3)	173(3)		O1W–H1W···O6	1.81(3)	2.662(6)	156(7)
O1W–H2W···O2	1.814(2)	2.669(3)	163(3)		O1W–H2W···O9W	1.84(2)	2.708(7)	170(6)
O2W–H3W···O9^iv^	1.86(2)	2.738(3)	172(3)		O2W–H3W···O4^iii^	1.71(3)	2.603(7)	167(7)
O2W–H4W···O7^i^	1.99(2)	2.846(3)	161(3)		O2W–H4W···O9W^iv^	1.86(2)	2.762(8)	178(6)
O3W–H5W···O4^i^	1.82(2)	2.675(3)	160(4)		O3W–H5W···O3^iii^	1.80(3)	2.677(7)	165(6)
O3W–H6W···O8W^iv^	1.837(2)	2.722(3)	175(4)		O3W–H6W···O6W	1.82(3)	2.695(7)	161(6)
O4W–H7W···O8W	1.870(2)	2.745(3)	179(4)		O4W–H7W···O6W^vi^	1.90(4)	2.726(7)	153(7)
O4W–H8W···O8^v^	1.706(2)	2.603(3)	178(4)		O4W–H8W···O3^ii^	2.04(4)	2.846(7)	149(7)
O5W–H9W···O7^v^	1.80(2)	2.669(3)	169(3)		O5W–H9W···O7^i^	1.79(2)	2.661(6)	165(6)
O5W–H10W···O9W	1.83(2)	2.693(3)	167(3)		O5W–H10W···O3W^vii^	1.92(2)	2.807(7)	170(6)
O6W–H11W···O8	2.49(3)	3.021(3)	120(3)		O6W–H11W···O1	2.60(5)	3.124(7)	117(4)
O6W–H11W···O7W^vi^	2.13(2)	2.951(4)	155(3)		O6W–H11W···O2	1.83(2)	2.722(6)	167(6)
O6W–H12W···O4	1.840(2)	2.712(4)	171(4)		O6W–H12W···O3^v^	1.90(3)	2.757(6)	159(7)
O7W–H14W···O3W	1.88(2)	2.809(4)	171(4)		O7W–H13W···O1W	1.93(2)	2.833(8)	169(8)
O8W–H15W···O5	2.21(3)	2.961(3)	146(3)		O7W–H14W···O6^x^	2.47(4)	3.271(9)	146(7)
N1–H1···O6^vii^	1.96(2)	2.808(4)	162(3)		O9W–H17W···O1^vi^	2.12(4)	2.983(7)	160(7)
N1–H2···O6W^vii^	1.920(2)	2.797(4)	176(3)		O9W–H18W···O8W	1.95(3)	2.790(8)	155(7)
N1–H3···O2^vii^	1.95(2)	2.784(4)	158(3)		O8W–H15W···O4^ix^	2.59(9)	3.025(8)	124(9)
N2–H10···O6^vii^	2.55(3)	3.174(4)	130(3)		O8W–H15W···O7W	2.28(9)	2.922(9)	156(11)
O8W–H16W···O6W^viii^	1.92(2)	2.777(4)	162(4)		O8W–H16W···O6^x^	1.90(8)	2.707(8)	172(8)
O9W–H17W···O7^vi^	1.881(2)	2.755(3)	170(3)		N1–H1···O7W	2.34(4)	3.048(9)	149(7)
O9W–H18W···O5	2.56(3)	3.130(3)	123(3)		N1–H2···O2i^x^	2.54(7)	3.183(8)	143(7)
O9W–H18W···O6	1.865(2)	2.742(3)	172(3)		N2–H2A···O7^vii^	2.10	2.776(7)	130
N2–H9···O7W	2.28(3)	3.041(4)	147(3)		N2–H2B···O8Wi^i^	2.26	2.799(8)	117

Symmetry transformations used to generate equivalent atoms: **SmCP1** data: (i) *−x+3/2, y−1/2, −z+3/2; (ii) *−x+3/2, y+1/2, −z+3/2;** (iii) *−x+1, −y, −z+1;* (iv) *x+1, y, z;* (v) *−x+1/2, y−1/2, −z+3/2;* (vi) *x−1/2, −y+1/2, z−1/2;* (vii) *x+1/2, −y+1/2, z+1/2;* **TbCP1** data: (i) *−x+3/2, y−1/2, −z+3/2;* (ii) *−x+3/2, y+1/2, −z+3/2;* (iii) *−x+5/2, y+1/2, −z+3/2;* (iv) *x+1, y, z;* (v) *x+1/2, −y+1/2, z+1/2;* (vi) *x−1, y, z;* (vii) *−x+2, −y+1, −z+2;* (viii) *−x+2, −y+1, −z+1;* (ix) *x−1/2, −y+1/2, z −1/2;* (x) *−x+1, −y+1, −z+1*.

**Table 4:** Geometric information (distances in Å and angles in degrees) for the D–H···O hydrogen bond interactions of the **TbCP2**, {[Tb(Haip)(aip)(H_2_O)_2_]·H_2_O}*_n_*, crystal structure.

D–H···O	*d* (H···O)	*d* (D···O)	∠ (DHO)		D–H···O	*d* (H···O)	*d* (D···O)	∠ (DHO)
O1W−H2W···O6^v^	2.01(4)	2.759(4)	164(5)		N1−H3N···O7^ix^	2.17(4)	2.717(4)	123(4)
O2W−H3W···O8^vi^	2.14(2)	3.024(4)	167(4)		N2−H4N···O3W^viii^	2.354(19)	3.193(6)	171(4)
O2W−H4W···O3W	1.994(19)	2.873(5)	170(4)		N2−H5N···O5^viii^	2.26(2)	3.106(4)	174(4)
N1−H1N···O2^vii^	1.90(3)	2.719(4)	154(4)		O3W−H5W···O1	2.13(2)	2.985(5)	157(5)
N1−H2N···O6^viii^	1.80(2)	2.687(4)	172(4)					

Symmetry transformations used to generate equivalent atoms: (v) *x, −y+1/2, z−1/2*; (vi) *x−1, y, z*; (vii) *−x+1, −y+1, −z*; (viii) *−x+1, y+1/2, −z+1/2*; (ix) *x+1, y, z*.

**Table 5:** Geometric information (distances in Å and angles in degrees) for the D–H···O hydrogen bond interactions found in the crystal structure of **TbCP3**, [Tb(Haip)(aip)(phen)]·H_2_O.

D–H···O	*d* (H···O)	*d* (D···O)	∠ (DHO)
N1−H1N···O1W^v^	2.18(4)	2.984(8)	156(7)
N1−H2N···O1^vi^	2.06(3)	2.888(7)	162(7)
N2−H2A···O1W^vii^	2.36	3.085(8)	136.1
N2−H2C···O8^vii^	1.77	2.682(3)	176.9
O1W−H1W···O7	1.87(2)	2.764(7)	167(7)

Symmetry transformations used to generate equivalent atoms: (v) *−x, −y+1, −z;* (vi) *x+1, −y+3/2, z+1/2*; (vii) *−x+1, y+1/2, −z+1/2*.

**Table 6:** PL peak wavelengths and PL decay parameters of all the CPs prepared by HT and MWAS.

CP	Synthesis Method	λ_em_/nm	τ_x_/μs (%)^(a)^	τ_total_^(b)^/µs	X^2(c)^
SmCP1	HT	590 (max), 483, 493, 518, 560, 565, 605, 642, 649, 698	4.43 (100)	4.43	1.015
	MWAS	596 (max), 482, 492, 519, 559, 565, 605, 642, 648, 697	4.39 (100)	4.39	1.000
TbCP1	HT	492, 545 (max), 551, 583, 589, 622, 647, 668, 680	136.121 (50.14) 43.82 (10.09) 283.94 (39.76)	185.57	1.001
	MWAS	492, 545 (max), 550, 584, 589, 622, 648, 668, 680	278.43 (27.95) 121.331 (50.30) 43.662 (21.24) 5.13 (0.50)	148.15	1.004
TbCP2	HT	489, 543 (max), 548, 588, 620, 647	137.911 (29.53) 64.737 (57.72) 21.626 (12.76)	80.85	1.049
TbCP3	HT	490, 546 (max), 586, 622, 650	696.22 (76.77) 0.771 (0.25) 25.186 (15.96) 120.87 (7.02)	546.99	0.975

^a^ PL decay measured at the maximum emission peaks of each material. ^b^ Total lifetime (τ_total_) of each material is given by intensity weighted lifetime. ^c^ **X^2^** indicates the goodness of the fitting results.

## Data Availability

CIF files have been deposited with the Cambridge Crystallographic Data Centre as supplementary publication numbers CCDC-2064873 (**SmCP1**), CCDC-2064876 (**TbCP1**), CCDC-2064875 (**TbCP2**) and CCDC-2064874 (**TbCP3**). Copies of these data can be obtained online, https://www.ccdc.cam.ac.uk/structures/. Remaining data utilized and shown in this research publication are available upon request directly to the authors.

## Supplementary Materials

The following are available online at https://www.mdpi.com/article/10.3390/ma14071786/s1: detailed description of the CPs preparation and a figure of the crystalline solids obtained (Figure S1), additional figures and tables related with the crystal structures of all the CPs (Figures S2–S5, and Tables S1–S4), FT-IR spectra for all the CPs isolated by HT and MWAS (Figure S6), other results of the PL studies, particularly the PL excitation spectra (Figure S7) and graphical representation of PL intensity in function of lifetime (Figure S8).
