# Supplementary material for: Multidimensional Ln-Aminophthalate Photoluminescent Coordination Polymers
Source: Materials (Basel). 2021 Apr 4;14(7):1786. doi: 10.3390/ma14071786 (PMC8038553; doi:10.3390/ma14071786)
Supplement: Supplementary file 1 [file materials-14-01786-s001.pdf]

materials

Supplementary Material

Multidimensional Ln-Aminophthalate Photoluminescent Coordination Polymers

Carla Queirós [1], Chen Sun [2], Ana M. G. Silva [1], Baltazar de Castro [1], Juan Cabanillas-Gonzalez [2,*] and Luís Cunha-Silva [1,*]

[1] LAQV/REQUIMTE & Department of Chemistry and Biochemistry, Faculty of Sciences, University of Porto, 4169-007 Porto, Portugal; carla.queiros@fc.up.pt (C.Q); ana.silva@fc.up.pt (A.M.G.S.); bcastro@fc.up.pt (B.d.C.)
[2] Madrid Institute for Advanced Studies, IMDEA Nanociencia, Calle Faraday 9, Ciudad Universitaria de Cantoblanco, 28049, Madrid, Spain; chen.sun@imdea.org
* Correspondence: l.cunha.silva@fc.up.pt (L.C.-S.) Tel.:+351-22-040-2653; juan.cabanillas@imdea.org (J.C.-G) Tel.:+34-912998784

Citation: Queirós, C.; Sun, C.; Silva, A.M.G.; de Castro, B.; Cabanillas-Gonzalez, J.; Cunha-Silva, L. Multidimensional Ln-aminophthalate photoluminescent coordination polymers. *Materials* **2021**, *14*, 1786. https://doi.org/10.3390/ma14071786

Academic Editor: Andrei S. Potapov

Received: 24 February 2021
Accepted: 1 April 2021
Published: 4 April 2021

Publisher's Note: MDPI stays neutral with regard to jurisdictional claims in published maps and institutional affiliations.

Copyright: © 2021 by the authors. Licensee MDPI, Basel, Switzerland. This article is an open access article distributed under the terms and conditions of the Creative Commons Attribution (CC BY) license (http://creativecommons.org/licenses/by/4.0/).

1. Coordination Polymers Preparation

1.1. SmCP1

Two synthetic procedures were used in the preparation of **SmCP1**, a hydrothermal synthesis and MWAS:

a) **Hydrothermal synthesis**: a mixture of 5–aminoisophthalic acid (H_2aip, 1.0 equiv, 0.30 mmol, 54.1 mg), $SmCl_3{\cdot}6H_2O$ (0.68 equiv, 0.20 mmol, 74.5 mg), NaOH (1.1 equiv, 0.33 mmol, 13.3 mg) and water (5 mL) was prepared in a 23 mL reaction vessel. The mixture was stirred at room temperature for 30 min and then the reaction vessel was sealed and heated to 110ºC for 72 h. After cooling, a mixture of white and light brown solids was filtered, separated, washed with water and dried at air. The brown crystalline solid corresponded to **SmCP1**; 27% yield was obtained (53.7 mg).

b) **MWAS**: a mixture of H_2aip (1.0 equiv, 0.30 mmol, 55.5 mg), $SmCl_3{\cdot}6H_2O$ (0.67 equiv, 0.20 mmol, 73.3 mg), NaOH (1.3 equiv, 0.39 mmol, 15.5 mg) and water (5.0 mL) was stirred for 30 min in a closed 10 mL microwave vessel. The vessel was then placed in the cavity of a microwave reactor. The reaction mixture was irradiated at 383 K (1 min ramp to 383 K and 2 h at 383 K, using 90 W maximum power and the mixture was maintained with stirring). After cooling, the resulting mixture was filtered and the filtrate was left standing at room temperature. Brown crystals, suitable for SCXRD, were obtained from the solid and from the filtrate (crystals were collected after 24 h) then dried at room temperature; 21% yield (43.1 mg).

ATR–FTIR: 3296 w, 2834 s, 2628 s, 1622 m, 1530 m, 1470 s, 1358 w, 1290 s, 1140 s.

1.2. TbCP1

As for **SmCP1**, hydrothermal and MWAS procedures were utilized in the preparation of **TbCP1**:

a) Hydrothermal synthesis: H_2aip (1.0 equiv., 0.31 mmol, 55.4 mg), $TbCl_3.6H_2O$ (0.66 equiv., 0.20 mmol, 75.2 mg), and NaOH (1.2 equiv., 0.39 mmol, 15.6 mg) were added to water (5 mL) and transferred to a 23 mL reaction vessel. The solution was stirred at room temperature for 30 min. Two small additions of NaOH (2.0 M) were performed until 3< pH <4, and then the reaction vessel was sealed and heated to 110 °C for 72 h. After cooling a mixture of white and light brown solids was filtered, separated by hand, washed with water and let to dry. The brown crystalline solid corresponded to **TbCP1**; 37% yield (77.8 mg).

b) MWAS: a mixture of H_2aip (1.0 equiv, 0.30 mmol, 54.7 mg), $TbCl_3.6H_2O$ (0.66 equiv., 0.20 mmol, 74.2 mg) and NaOH (1.0 equiv., 0.32 mmol, 12.7 mg) and water (5.0 mL) was stirred for 30 min in a closed 10 mL microwave vessel. After that time period the

vessel was placed in the cavity of a microwave reactor. The reaction mixture was irradiated at 383 K (1 min ramp to 110 °C and 6 h at 110 °C, using 90 W maximum power and the mixture was maintained without stirring). After cooling the resulting mixture filtered and the filtrate was left standing at r.t. Brown crystals suitable for SCXRD were obtained from the filtered solid and dried at r.t. (16% yield, 29.6 mg).

ATR-FTIR: 3280 w, 2944 s, 2826 w, 2634 s, 2362 s, 1622 m, 1536 m, 1468 m, 1440 s, 1370 m, 1288 m, 1142 s, 998 s, 942 s, 766 m.

1.3. *TbCP2*

A hydrothermal procedure was utilized in the preparation of **TbCP2**: a mixture of H_2aip (1.0 equiv., 0.30 mmol, 54.8 mg), $TbCl_3.6H_2O$ (0.66 equiv., 0.20 mmol, 74.7 mg), and NaOH (1.0 equiv., 0.31 mmol, 12.3 mg) was added to water (5 mL) and transferred to a 23 mL reaction vessel. The solution was stirred at room temperature for 30 min, and then the reaction vessel was sealed and heated to 110 °C for 72 h. After cooling brown crystals were filtered, washed with water and acetone and let to dry; 27% yield (46.9 mg).

ATR-FTIR: 3238 w, 2958 w, 2610 s, 2364 m, 2338 m, 1916 m, 1698 s, 1626 s, 1538 m, 1456 s, 1398 m, 1284 s, 1220 s, 1114 s, 902 s, 750 m, 596 s, 550 s, 500 s.

1.4. *TbCP3*

A hydrothermal procedure was utilized in the preparation of **TbCP3**: H_2aip (1.0 equiv., 0.30 mmol, 54.9 mg), $TbCl_3.6H_2O$ (0.66 equiv., 0.20 mmol, 74.5 mg), *phen* (0.59 equiv., 0.18 mmol, 35.0 mg) and NaOH (1.2 equiv., 0.36 mmol, 14.6 mg) were added to water (5 mL) and transferred to a 23 mL reaction vessel. The solution was stirred at room temperature for 30 min, and then the reaction vessel was sealed and heated to 110 °C for 72 h. After cooling a yellow/brown crystals were filtered, washed with water and acetone and dried at air; 47% yield (60.3 mg).

ATR-FTIR: 3616 s, 3356 s, 3054 s, 1644 m, 1596 m, 1550 m, 1516 m, 1470 s, 1452 s, 1394 m, 128 m, 1254 m, 1140, 1100 s, 1000 s, 912 s, 864 s, 842 m, 800 m, 776 m, 760 s, 722 m, 694 m, 625 s, 566 s, 498 s.

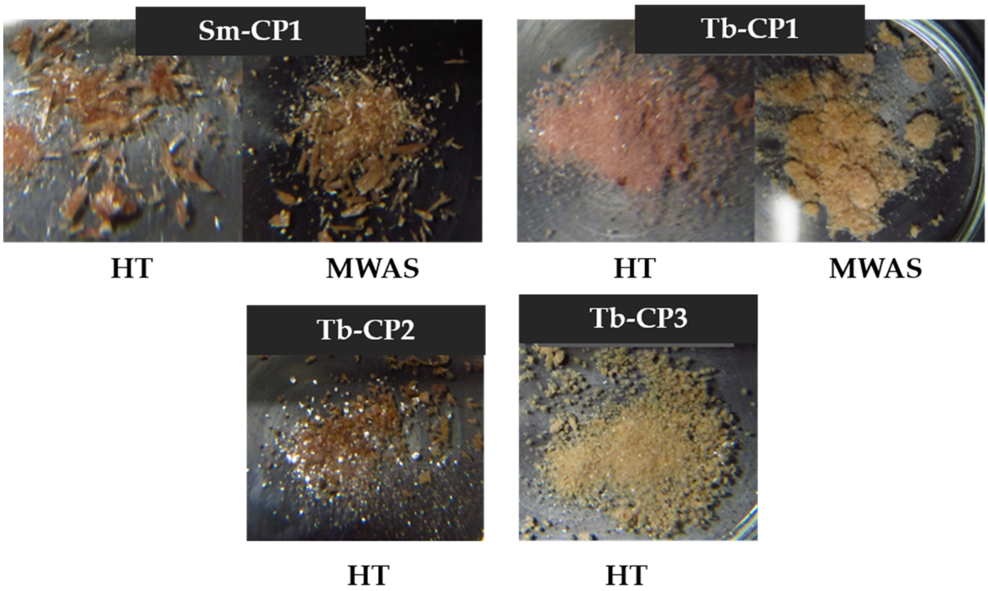

Figure S1. Crystalline solids of **SmCP1** and **TbCP1** (prepared by hydrothermal—HT and MWAS), **TbCP2** and **TbCP3** (prepared by HT synthesis); solids were obtained after filtration and drying at room temperature).

2. Crystal Structures

2.1. SmCP1

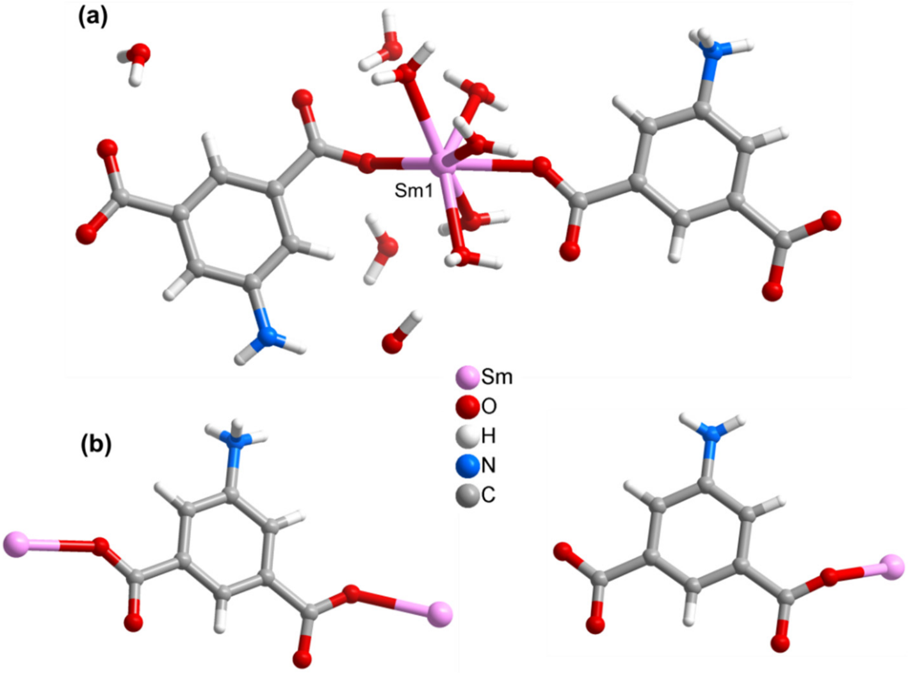

Figure S2. (**a**) The asymmetric unit (asu) of the crystal structure of 1D CP **SmCP1**, **{[Sm(Haip)(aip)(H$_2$O)$_5$]·4(H$_2$O)}$_n$**, represented in the ball and stick model. (**b**) Coordination modes of the ligands Haip$^-$ (left side) and aip^{2-} (right side).

Table S1. Selected distances and angles of the Sm^{3+} coordination centre of the material **SmCP1**, **{[Sm(Haip)(aip)(H$_2$O)$_5$]·4(H$_2$O)}$_n$**.

Distance/Å		Angles/°			
Sm1–O3^i	2.371(2)	O3^i–Sm1–O5	139.81(7)	O4W–Sm1–O1W	142.37(8)
Sm1–O5	2.390(2)	O3^i–Sm1–O1	140.49(7)	O3^i–Sm1–O3W	78.50(8)
Sm1–O1	2.401(2)	O5–Sm1–O1	78.05(7)	O5–Sm1–O3W	111.36(7)
Sm1–O2W	2.428(2)	O3^i–Sm1–O2W	70.87(8)	O1–Sm1–O3W	75.24(8)
Sm1–O4W	2.428(2)	O5–Sm1–O2W	148.20(8)	O2W–Sm1–O3W	77.77(8)
Sm1–O1W	2.430(2)	O1–Sm1–O2W	75.07(8)	O4W–Sm1–O3W	72.43(8)
Sm1–O3W	2.456(2)	O3^i–Sm1–O4W	72.62(8)	O1W–Sm1–O3W	144.43(8)
Sm1–O5W	2.471(2)	O5–Sm1–O4W	73.82(8)	O3^i–Sm1–O5W	71.06(7)
		O1–Sm1–O4W	124.77(8)	O5–Sm1–O5W	81.88(7)
		O2W–Sm1–O4W	136.47(8)	O1–Sm1–O5W	140.96(7)
		O3^i–Sm1–O1W	113.60(7)	O2W–Sm1–O5W	109.20(8)
		O5–Sm1–O1W	81.58(7)	O4W–Sm1–O5W	79.86(8)
		O1–Sm1–O1W	75.48(7)	O1W–Sm1–O5W	68.67(8)
		O2W–Sm1–O1W	75.52(8)	O3W–Sm1–O5W	143.71(8)

Symmetry transformation used to generate equivalent atoms: (i) *−x+3/2, y−1/2, −z+3/2;*

2.2. *TbCP1*

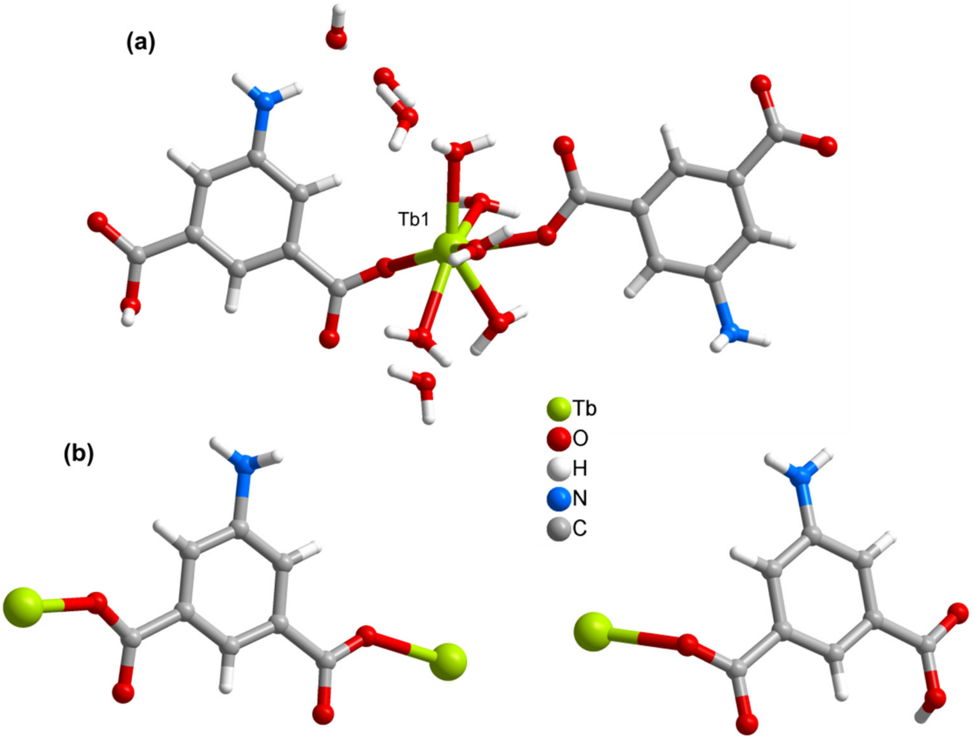

Figure S3. (**a**) The asymmetric unit (asu) of the crystal structure of 1D CP **TbCP1**, **{[Tb(Haip)(aip)(H_2O)$_5$]·4(H_2O)}$_n$**, represented in the ball–and–stick model. (**b**) Coordination modes of the ligands aip^{2-} (left side) and Haip^{-} (right side).

Table S2. Selected distances and angles of the Tb^{3+} coordination centre of the 1D CP **TbCP1**, **{[Tb(Haip)(aip)(H_2O)$_5$]·4(H_2O)}$_n$.**

Distance/Å		Angles/°			
Tb1–O5	2.338(4)	O5–Tb1–O1	140.18(17)	O5W–Tb1–O2W	142.57(18)
Tb1–O1	2.339(5)	O5–Tb1–O8^{i}	140.39(17)	O5–Tb1–O1W	78.95(16)
Tb1–O8^{i}	2.365(4)	O1–Tb1–O8^{i}	77.62(16)	O1–Tb1–O1W	110.53(16)
Tb1–O4W	2.381(5)	O5–Tb1–O4W	70.73(17)	O8^{i}–Tb1–O1W	74.55(15)
Tb1–O5W	2.390(5)	O1–Tb1–O4W	148.00(16)	O4W–Tb1–O1W	78.41(19)
Tb1–O2W	2.392(5)	O8^{i}–Tb1–O4W	75.42(17)	O5W–Tb1–O1W	144.77(17)
Tb1–O1W	2.407(5)	O5–Tb1–O5W	113.51(16)	O2W–Tb1–O1W	71.73(18)
Tb1–O3W	2.427(5)	O1–Tb1–O5W	81.51(16)	O5–Tb1–O3W	71.34(16)
		O8^{i}–Tb1–O5W	76.18(15)	O1–Tb1–O3W	81.91(16)
		O4W–Tb1–O5W	75.59(18)	O8^{i}–Tb1–O3W	141.25(15)
		O5–Tb1–O2W	72.88(17)	O4W–Tb1–O3W	109.31(18)
		O1–Tb1–O2W	73.97(17)	O5W–Tb1–O3W	68.45(16)
		O8i–Tb1–O2W	123.79(17)	O2W–Tb1–O3W	80.28(17)
		O4W–Tb1–O2W	136.38(17)	O1W–Tb1–O3W	144.01(16)

Symmetry transformation used to generate equivalent atoms: (i) $x+3/2$, $y-1/2$, $-z+3/2$

2.3. TbCP2

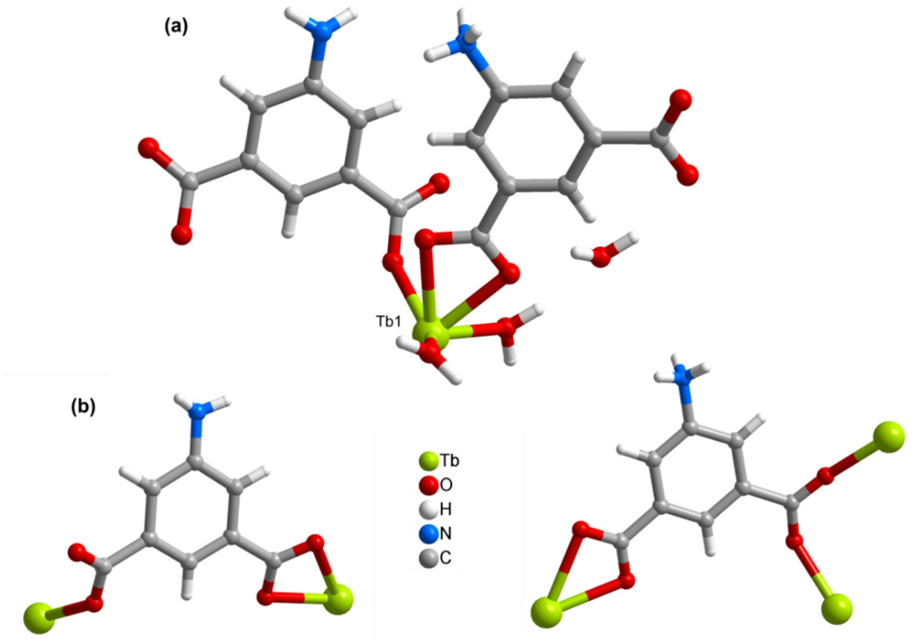

Figure S4. (**a**) The asymmetric unit (asu) of the crystal structure of 2D CP **TbCP2**, {[Tb(Haip)(aip)(H_2O)$_2$]·H_2O}$_n$, represented in the ball–and–stick model. (**b**) Coordination modes of the ligands aip^{2-} (left side) and Haip^{-} (right side).

Table 3. Selected distances and angles of the Tb^{3+} coordination centre of the 2D CP **TbCP2**, {[Tb(Haip)(aip)(H_2O)$_2$]·H_2O}$_n$.

Distance/Å		Angles/°			
Tb1–O3^{i}	2.290(3)	O3^{i}–Tb1–O4^{ii}	92.91(9)	O7^{iii}–Tb1–O2W	148.00(10)
Tb1–O4^{ii}	2.297(2)	O3^{i}–Tb1–O5	155.01(10)	O1–Tb1–O2W	70.00(10)
Tb1–O5	2.376(3)	O4^{ii}–Tb1–O5	75.78(9)	O1W–Tb1–O2W	135.44(11)
Tb1–O7^{iii}	2.402(3)	O3^{i}–Tb1–O7^{iii}	125.23(9)	O3^{i}–Tb1–O2	122.29(8)
Tb1–O1	2.455(3)	O4^{ii}–Tb1–O7^{iii}	84.01(9)	O4^{ii}–Tb1–O2	143.97(9)
Tb1–O1W	2.466(3)	O5–Tb1–O7^{iii}	76.41(9)	O5–Tb1–O2	68.81(8)
Tb1–O2W	2.521(3)	O3^{i}–Tb1–O1	81.52(9)	O7^{iii}–Tb1–O2	81.50(8)
Tb1–O2	2.583(3)	O4^{ii}–Tb1–O1	138.86(10)	O1–Tb1–O2	51.41(9)
Tb1–O8^{iii}	2.661(3)	O5–Tb1–O1	92.37(9)	O1W–Tb1–O2	68.76(10)
		O7^{iii}–Tb1–O1	131.98(9)	O2W–Tb1–O2	109.91(9)
		O3^{i}–Tb1–O1W	70.41(10)	O3^{i}–Tb1–O8^{iii}	76.93(9)
		O4^{ii}–Tb1–O1W	138.40(10)	O4^{ii}–Tb1–O8^{iii}	68.43(9)
		O5–Tb1–O1W	132.14(10)	O5–Tb1–O8^{iii}	117.58(9)
		O7^{iii}–Tb1–O1W	76.44(10)	O7^{iii}–Tb1–O8^{iii}	51.11(8)
		O1–Tb1–O1W	77.84(10)	O1–Tb1–O8^{iii}	146.31(9)
		O3^{i}–Tb1–O2W	74.95(11)	O1W–Tb1–O8^{iii}	70.70(10)
		O4^{ii}–Tb1–O2W	69.20(10)	O2W–Tb1–O8^{iii}	127.01(9)
		O5–Tb1–O2W	80.18(10)	O2–Tb1–O8^{iii}	123.08(9)

Symmetry transformations used to generate equivalent atoms: (i) $-x, -y+1, -z$; (ii) $x, y-1, z$; (iii) $-x+1, -y, -z$; (iv) $x, y+1, z$.

2.4. *TbCP3*

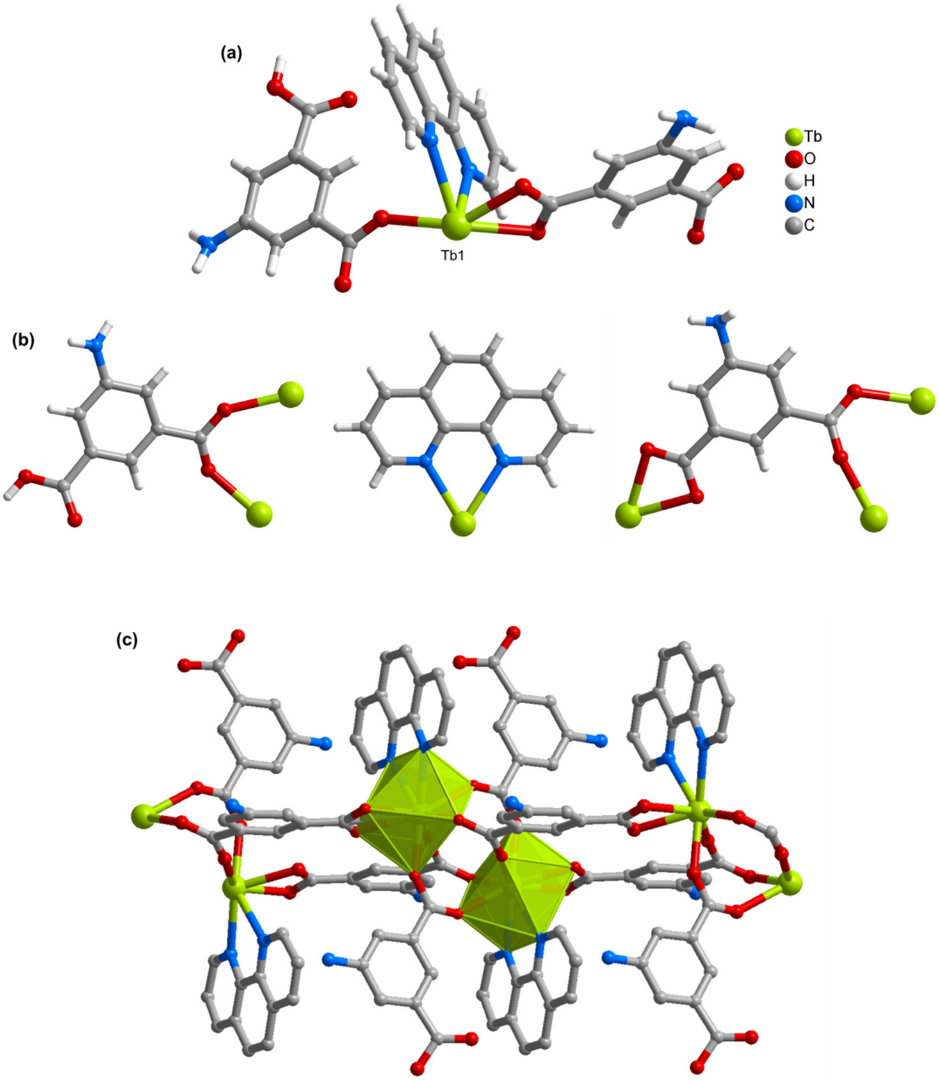

Figure S5. (**a**) *asu* of the crystal structure of 1D CP **TbCP3**, {**[Tb(Haip)(aip)(phen)]·(H$_2$O)**}$_n$, represented in the ball–and–stick model. (**b**) Coordination modes of the ligands Haip$^-$ (left side), phen (centre) and aip^{2-} (right side).

Table S4. Selected distances and angles of the Tb^{3+} coordination centre of the coordination chain material **TbCP3**, {**[Tb(Haip)(aip)(phen)]·(H$_2$O)**}$_n$.

Distance/Å		Angles/°			
Tb1–O3^{i}	2.276(4)	O(3)^{i}–Tb(1)–O(6)^{ii}	78.64(15)	O(1)–Tb(1)–O(2)	53.79(12)
Tb1–O6^{ii}	2.302(4)	O(3)^{i}–Tb(1)–O(5)	76.68(15)	O(3)^{i}–Tb(1)–N(3)	85.99(15)
Tb1–O5	2.310(4)	O(6)^{ii}–Tb(1)–O(5)	125.61(14)	O(6)^{ii}–Tb(1)–N(3)	144.67(16)
Tb1–O4^{iii}	2.335(4)	O(3)^{i}–Tb(1)–O(4)^{iii}	124.93(14)	O(5)–Tb(1)–N(3)	80.17(15)
Tb1–O1	2.425(4)	O(6)^{ii}–Tb(1)–O(4)^{iii}	75.50(14)	O(4)^{iii}–Tb(1)–N(3)	137.72(15)
Tb1–O2	2.452(4)	O(5)–Tb(1)–O(4)^{iii}	80.40(15)	O(1)–Tb(1)–N(3)	85.48(15)
Tb1–N3	2.563(5)	O(3)^{i}–Tb(1)–O(1)	131.63(14)	O(2)–Tb(1)–N(3)	71.25(15)
Tb1–N4	2.598(5)	O(6)^{ii}–Tb(1)–O(1)	81.53(14)	O(3)^{i}–Tb(1)–N(4)	138.80(15)
		O(5)–Tb(1)–O(1)	147.30(13)	O(6)^{ii}–Tb(1)–N(4)	141.86(15)
		O(4)^{iii}–Tb(1)–O(1)	91.15(14)	O(5)–Tb(1)–N(4)	71.53(14)
		O(3)^{i}–Tb(1)–O(2)	78.44(14)	O(4)^{iii}–Tb(1)–N(4)	74.76(15)
		O(6)^{ii}–Tb(1)–O(2)	74.57(14)	O(1)–Tb(1)–N(4)	75.77(14)
		O(5)–Tb(1)–O(2)	143.08(14)	O(2)–Tb(1)–N(4)	113.36(14)
		O(4)^{iii}–Tb(1)–O(2)	136.50(13)	N(3)–Tb(1)–N(4)	63.54(16)

Symmetry transformations used to generate equivalent atoms: (i) $-x,-y+1,-z+1$; (ii) $-x+1, -y+1 -z+1$; (iii) $x+1, y, z$; (iv) $x-1, y, z$;

3. FT-IR Spectra

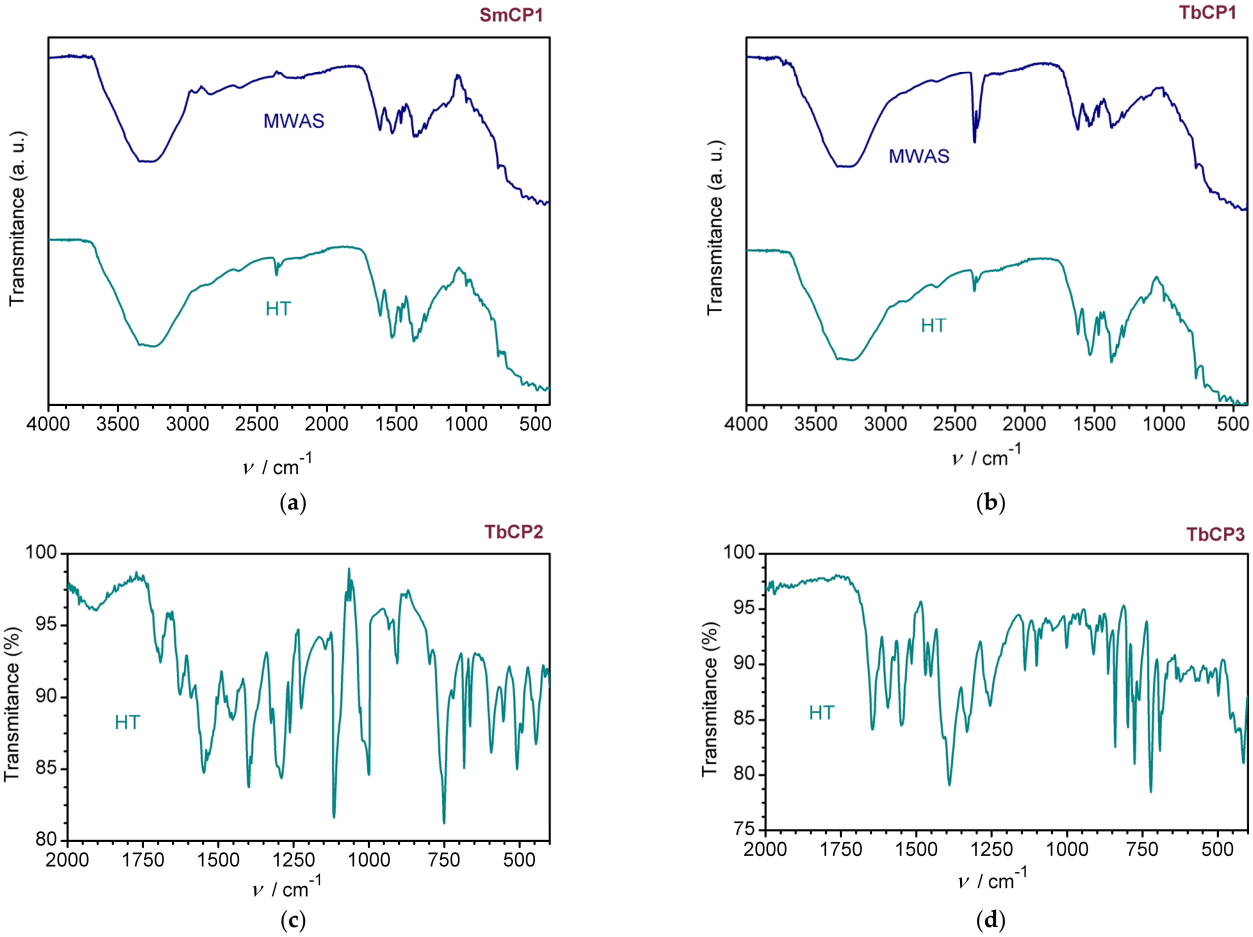

Figure 6. FTIR spectra of all the CP materials prepared and reported: SmCP1 (**a**), TbCP1 (**b**), TbCP2 (**c**) and TbCP3 (**d**).

4. PL Studies

4.1. PL Excitation Spectra

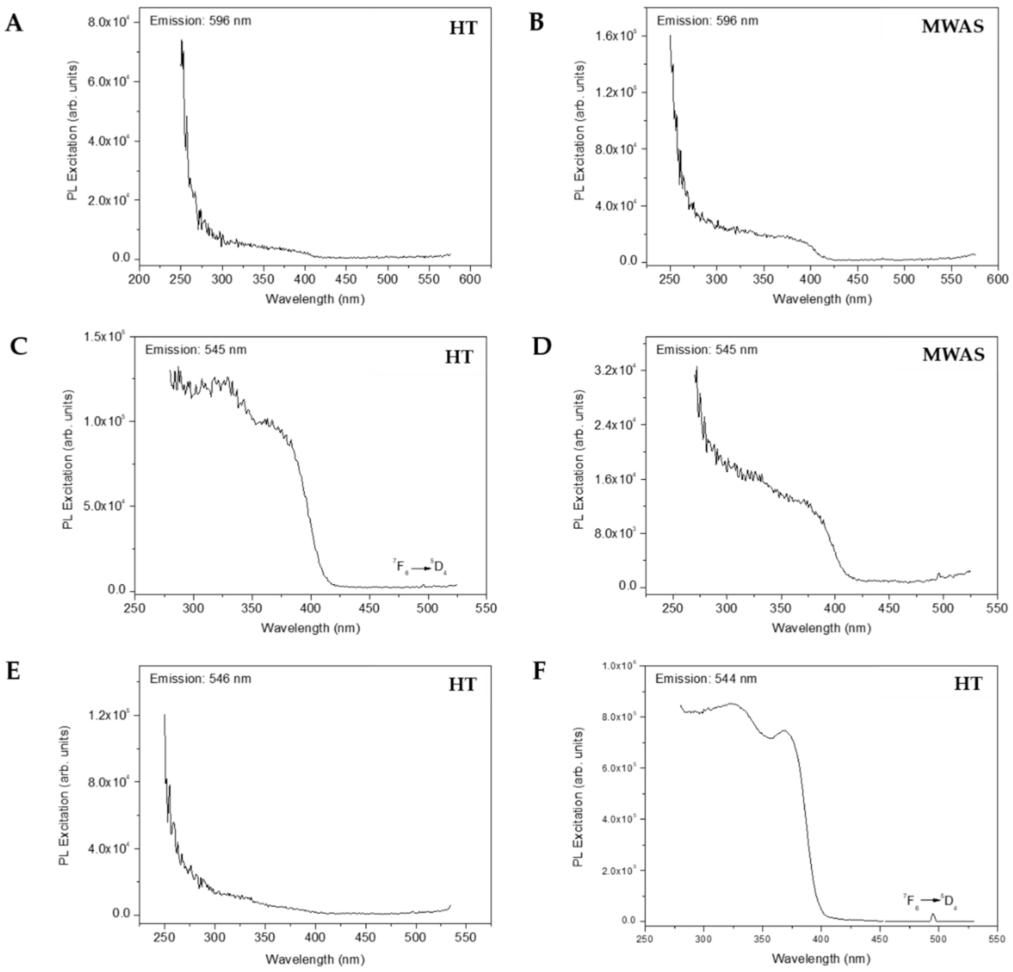

Figure 7. Graphical representations of PL excitation spectra of **SmCP1**, prepared by HT (A) and MWAS (B), **TbCP1**, prepared by HT (C) and MWAS (D), **TbCP2**, prepared by HT (E) and **TbCP3**, prepared by HT (F). The detection wavelengths of the PL excitation spectra were written in each subplots, respectively.

4.2. PL Intensity in Function of Lifetime

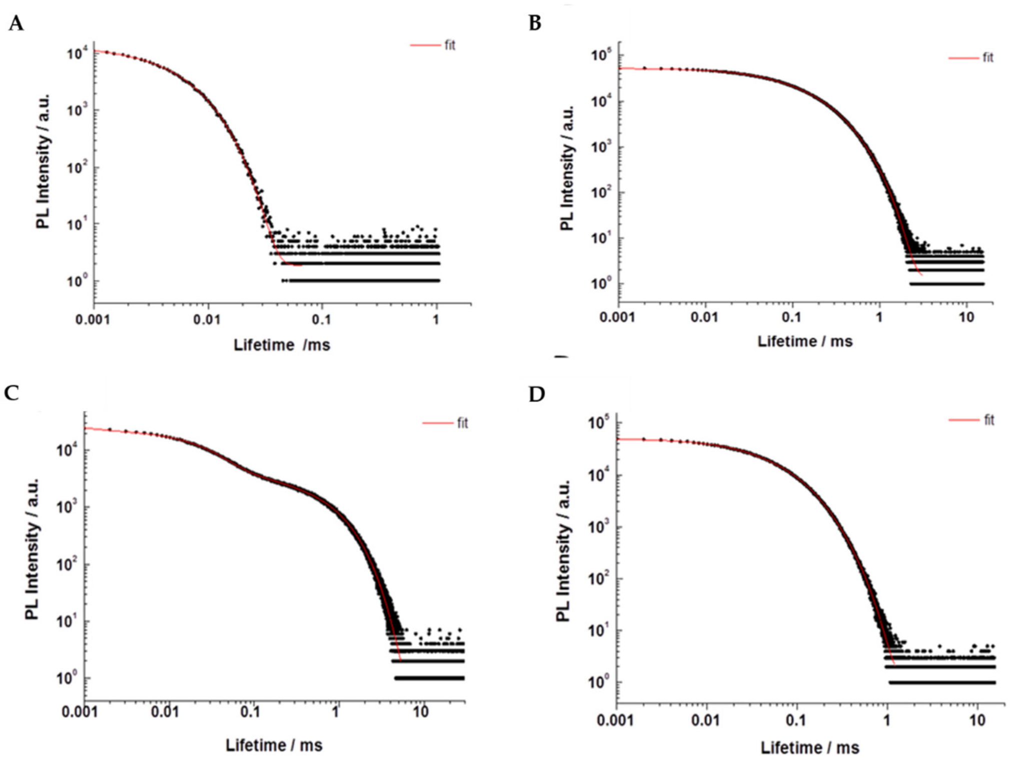

Figure 8. Graphical representations of the PL intensity as function of lifetime (black dots) for **SmCP1** (**A**), **TbCP1** (**B**), **TbCP2** (**C**) and **TbCP3** (**D**) together with their fittings (red lines). The rising tails below 300 nm in A, B, C, D and E are caused by excitation light scattering.
